# Evaluation of mitochondrial genomics in *Salmo trutta caspius*

**DOI:** 10.1080/23802359.2017.1365635

**Published:** 2017-09-13

**Authors:** Seddigheh Amini, Abolhasan Rezaei

**Affiliations:** Department of Genetics, Faculty of Biological Sciences, Islamic Azad University, Tonekabon Branch, Tonekabon, Iran

**Keywords:** *Salmo trutta caspius*, mitochondrial genomics

## Abstract

*Salmo trutta caspius* population is an important species for aquaculture and livestock industry. Moreover, these species are used for studies on molecular markers. Mitochondrial genomic is also beneficial for phylogenetic studies in salmonid species. They are applied for maternal traits whereas paternal traits are related to nuclear genomics. Mtgenomics will be genetically highly divergent indicating that they may represent distinct and potentially locally adapted gene pools. Evolutionary history of the salmo taxa such as brown trout, salmo salar, and *s. trutta* populations has been studied. In the present study, 50 samples of the Iranian *S.t. caspius* population were collected from three regions [Tonekabon (Cheshmekileh Roud), Ramsar (Safa Roud), and Talesh (Nav roud)]. The PCR product was carried out for machinery sequencing. Mtgenomic in *S.t. caspius* was deposited in GenBank under accession no. LC011387.1. Evolutionary analyses were conducted using MEGA7 software. The analysis involved 50 nucleotide sequences. A close relationship was observed between the samples.

The *Salmo trutta caspius* population is an important species for the aquaculture and livestock industry. Moreover, these species are used for studies on molecular markers. Mitochondrial genomic is also beneficial for phylogenetic studies on salmonid species. They are applied for maternal traits whereas paternal traits are related to nuclear genomics. Mitochondrial genomics are genetically highly divergent indicating that they may represent distinct and potentially locally adapted gene pools (Apostolidis et al. 2011). Previous researches have studied the evolutionary history of the *salmo* taxa species including *brown trout*, *s. salar*, and *s. trutta* populations (Nei 1987; Bernatchez and Wilson 1998; Avise 2000; Osinov and Lebedev 2000; Kottelat and Freyhof 2007).

In the present study, 50 samples of *s.t. caspius* were collected from three regions [Tonekabon (Cheshmekileh Roud), Ramsar (Safa Roud) in Mazandaran province and Talesh (Nav Roud) in Guillan province]. The distance between Cheshmekileh Roud and Safa Roud to NavRoud is approximately 249 km.

**Figure 1.**
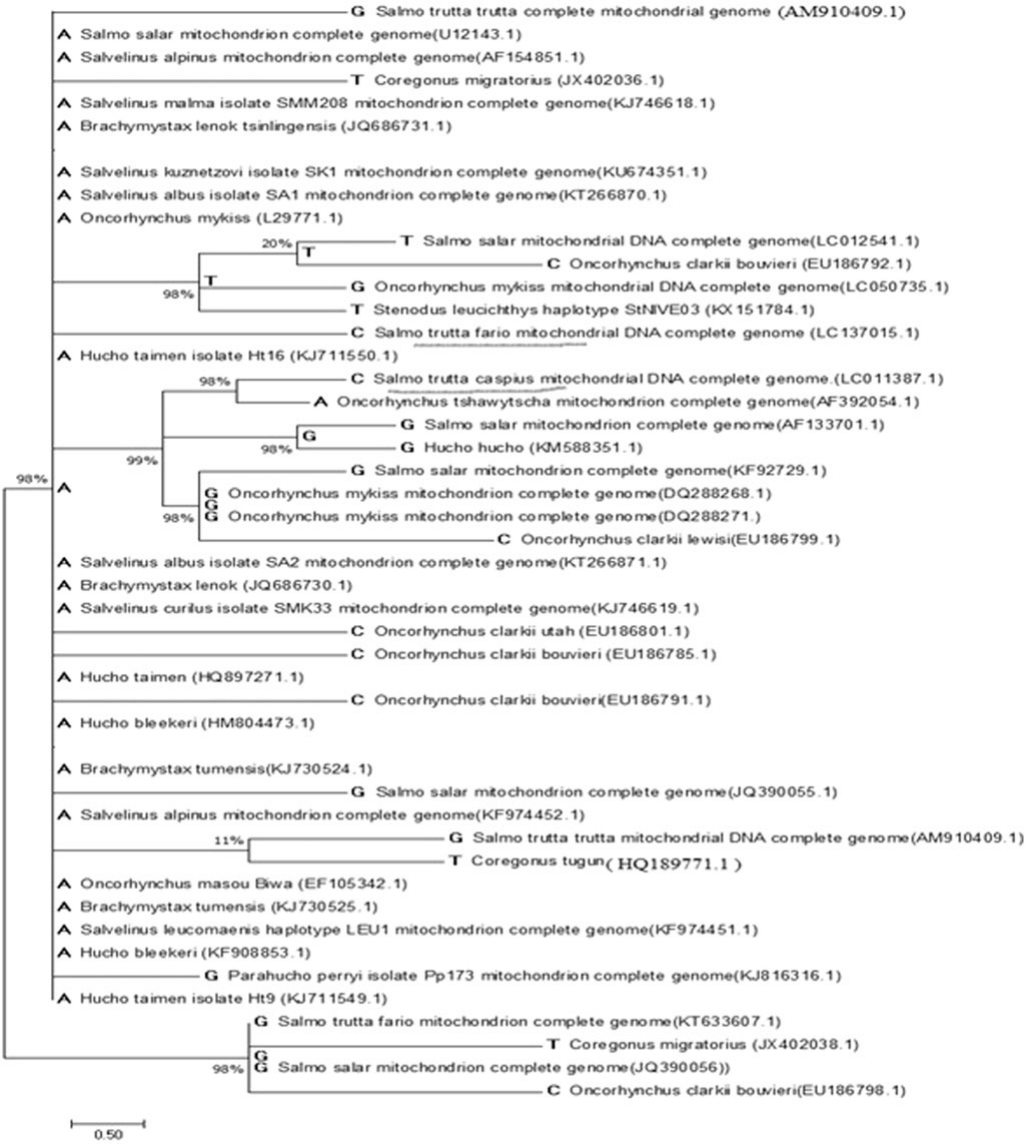
Ancestral states were inferred using the Maximum Parsimony method. The tree shows a set of possible nucleotides (states) at each ancestral node based on their inferred likelihood at Site 1. Ambiguous states are not shown. The initial tree was inferred using a pre-computed tree file. Evolutionary analyses were conducted using MEGA 7.0.

The sample salmonids caught in fall 2015 were three years old. For the next stage, total genomic DNA was isolated from blood taken from fresh specimens stored in −20 °C in the refrigerator. DNA isolation was carried out using the phenol–chloroform protocol (Sambrook and Russel 2001). After conducting the PCR technique, the universal primers were designed; thereby the PCR product was carried out for sequencing. Mtgenomic in *s.t. fario* was deposited in GenBank accession no. LC011387.1. Evolutionary analyses were conducted using MEGA Software Version 7 (Tamura and Nei 1993). The analysis involved 50 nucleotide sequences. There were 16,622 positions in the final dataset. Results showed a major diversity between *s.t. caspius* marked by black underline and other salmonids like *Hucho taimen, s.t. fario,* etc. A close relationship was observed between the samples (Figure 1).
